# Crossing the finish line towards a disease-modifying treatment for Angelman syndrome

**DOI:** 10.1186/s11689-026-09681-5

**Published:** 2026-03-07

**Authors:** Matthew C. Judson, Luis Pereira de Almeida, Rebecca D. Burdine, Stormy J. Chamberlain, Benjamin E. Deverman, Ben Distel, Michael D. Ehlers, Elizabeth Jalazo, Steven A. Kushner, Mark Nespeca, Stephan J. Sanders, Martin Scheffner, Jason J. Yi, Mark J. Zylka, Ype Elgersma, Benjamin D. Philpot

**Affiliations:** 1 Neuroscience Center, Department of Cell Biology & Physiology, Carolina Institute for Developmental Disabilities, University of North Carolina, Chapel Hill, NC USA; 2 Center for Neuroscience and Cell Biology (CNC-UC), Center for Innovative Biomedicine and Biotechnology (CIBB), Gene Therapy Center of Excellence (GeneT), Faculty of Pharmacy (FFUC), University of Coimbra, Coimbra, Portugal; 3 Department of Molecular Biology, Princeton University, Princeton, NJ USA; 4 Roche Pharma Research and Early Development, Neuroscience and Rare Disease discovery and translational area, Roche Innovation Center Basel, Basel, 4070 Switzerland; 5 Stanley Center for Psychiatric Research, Broad Institute of MIT and Harvard, Cambridge, MA 02142 USA; 6 Department of Clinical Genetics, Erasmus Medical Center, Erasmus MC Center of Expertise for Neurodevelopmental Disorders (ENCORE), Rotterdam, The Netherlands; 7 MPM BioImpact, Boston, MA USA; 8 Department of Pediatrics, Division of Genetics and Metabolism, University of North Carolina, Chapel Hill, NC USA; 9 Department of Psychiatry, SNF Center for Precision Psychiatry & Mental Health, Columbia University Irving Medical Center, Columbia University, New York, NY 10032 USA; 10 Departments of Neurosciences and Pediatrics, University of California San Diego and Rady Children’s Hospital San Diego, San Diego, CA USA; 11 Institute of Developmental and Regenerative Medicine, Department of Paediatrics, University of Oxford, Oxford, OX3 7TY UK; 12 Department of Psychiatry and Behavioral Sciences, UCSF Weill Institute for Neurosciences, University of California, San Francisco, San Francisco, CA 94158 USA; 13 Department of Biology, University of Konstanz, Konstanz, Germany; 14 Department of Neuroscience, Washington University School of Medicine, St. Louis, MO USA

## Abstract

Recent progress in the development of genetic therapies promises that impactful treatments for single-gene neurodevelopmental disorders are imminent. But can derailed neurodevelopmental processes be mended after broken genes are replaced or otherwise restored? The results of ongoing clinical trials for Angelman syndrome will soon yield answers to this pressing question, yet the trials face significant obstacles. Here we identify insights needed to aid the quest for a disease-modifying Angelman syndrome therapy, which could serve as a roadmap for the expeditious development of genetic therapies for other single-gene neurodevelopmental disorders.

Angelman syndrome (AS) is a neurodevelopmental disorder characterized by intellectual disability, seizures, ataxia, and lack of speech [1, 2]. Most (~70%) AS cases result from the deletion of ~20 genes in the maternally inherited 15q11-q13 chromosomal region [1]. However, loss-of-function mutations of the maternal *UBE3A* gene are sufficient to cause AS [3, 4], and thus, it is widely considered to be a monogenic disorder. Current treatments for AS are limited to symptom management, but transformative, disease-modifying therapeutics may soon be attainable (Figure 1). First, the paternally inherited *UBE3A* allele is a fortuitously tractable therapeutic target: paternal *UBE3A* is epigenetically inactivated in neurons by a long non-coding antisense transcript (*UBE3A-ATS*) [5, 6], but is otherwise intact and responsive to unsilencing by removing repression by *UBE3A-ATS*. Second, the *UBE3A* gene is amenable to packaging within clinically validated viral vectors for the purpose of gene replacement therapy [7, 8]. Third, *UBE3A* encodes an E3 ubiquitin ligase (known as UBE3A or E6-associated protein) that ubiquitinates and promotes the proteasomal degradation of substrate proteins, which themselves are potential targets for the development of adjunctive treatments to paternal *UBE3A* unsilencers or *UBE3A* gene therapies [9]. Last, but not least, despite penetrant mild-to-moderate microcephaly [10], there is little evidence of neurodegeneration or gross anatomical abnormalities in AS individuals that would hinder neurological recovery following treatment. Given this favorable therapeutic outlook, it is not surprising that multiple AS treatments are rapidly advancing to the clinic, with two, and soon to be three, phase 3 clinical trials already underway (NCT06914609, NCT06617429). With potential treatments on the horizon, we recently convened a panel of AS parents, preclinical experts, clinicians, and industry leaders with a shared goal of identifying gaps in our understanding of UBE3A function, AS biology, and the AS clinical landscape that, if left unbridged, could hamper the realization of these and yet-to-be discovered disease-modifying AS therapeutics. In the following synopsis of our discussions, we highlight the consensus unmet needs that are most essential for a clinical breakthrough in the treatment of AS, many of which are broadly applicable to other neurodevelopmental disorders (see Table 1).

**Fig. 1:**
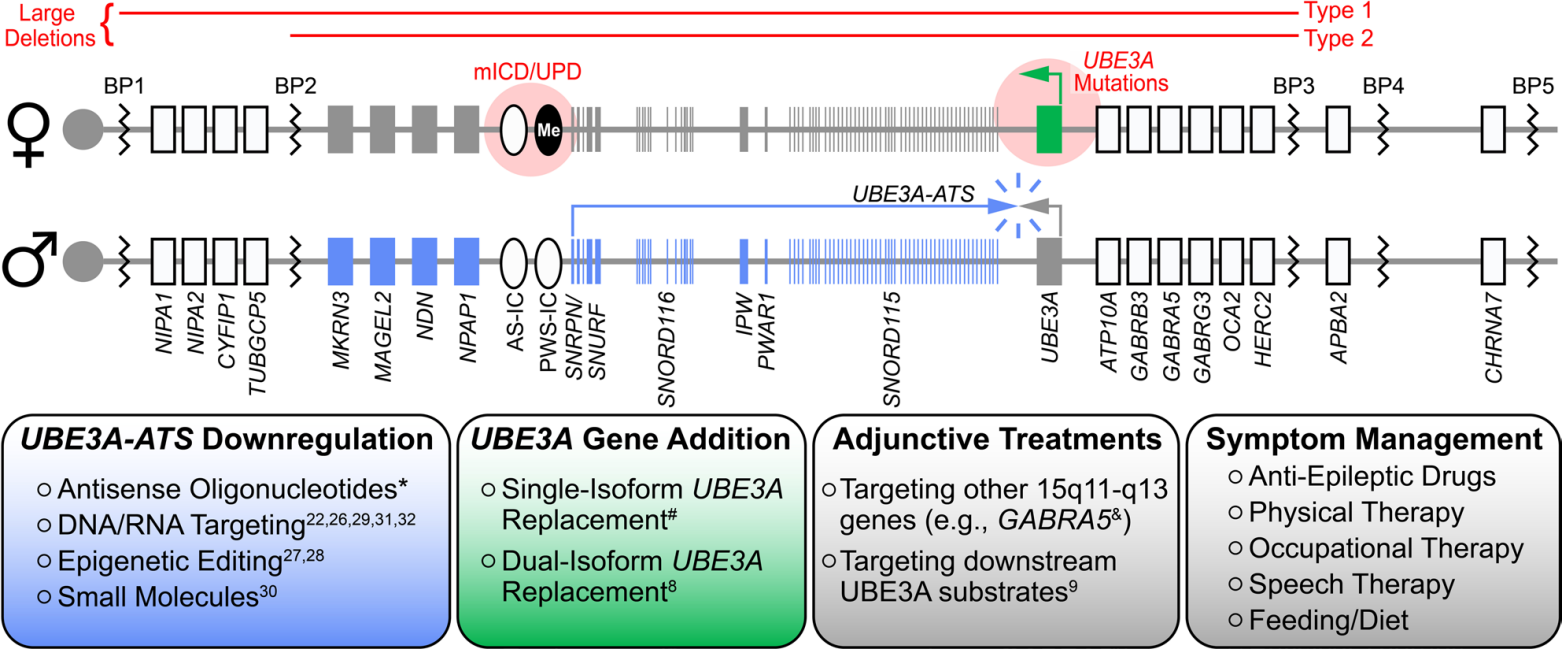
The therapeutic outlook for Angelman syndrome in 2026. Top: Schematics (not to scale) of the maternal (♀) and paternal (♂) 15q11-q13 chromosomal regions, highlighting genes with biallelic (white), paternal-only (blue), and maternal-only (green) expression in neurons. Red font/shading denotes common maternal deletions and mutations resulting in Angelman syndrome. Me: methylation. mICD: maternal imprinting center defect. UPD: paternal uniparental disomy. BP: breakpoint. Bottom: Key areas of therapeutic development and practice. *Clinical trials NCT06914609, NCT06617429, NCT07157254. ^#^Clinical trial NCT07181837. ^&^Clinical trial NCT05630066

**Table 1:**
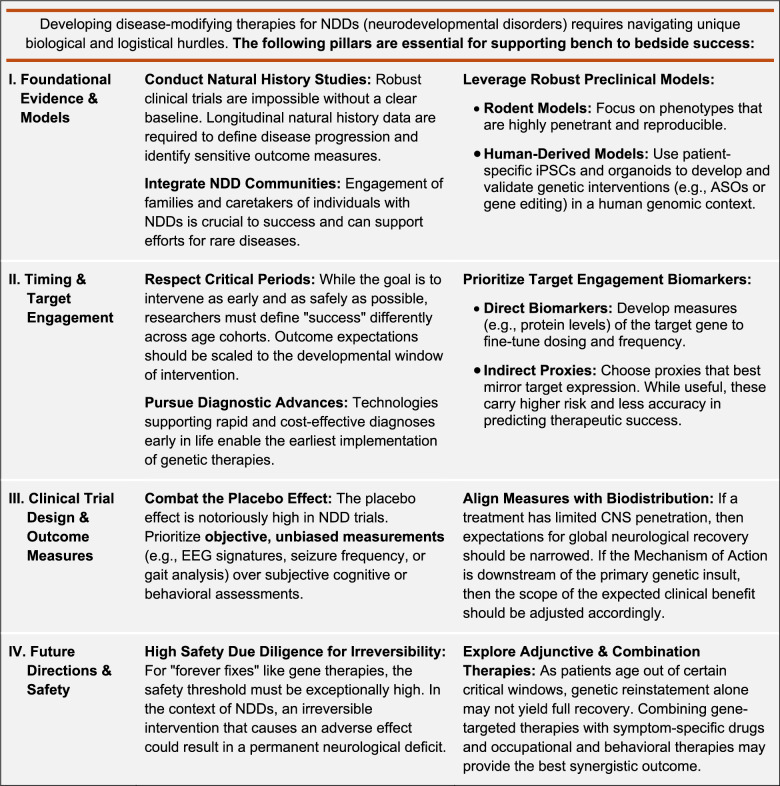
Key Considerations for Therapeutic Development in Angelman Syndrome and other NDDs

## Improved face validity for AS animal models

Since 1998, maternal *Ube3a*-deficient mice (i.e., AS model mice [11–13]) have been the bedrock of preclinical research campaigns geared toward elucidating the neurobiological underpinnings of AS pathophysiology. In the course of this work, penetrant behavioral phenotypes in AS mice have been identified in multiple laboratories: reduced motor performance in the rotarod task, hypoactivity in the open field test, reduced marble burying, reduced nest building, increased floating time in the swim/float task, enhanced kindling-induced epileptogenesis, and, in a strain-dependent manner, heightened susceptibility to audiogenic seizures [14, 15]. The remarkable reproducibility of these assays, established over many years and across multiple laboratories, has merited their frequent inclusion in preclinical test batteries to evaluate the efficacy of therapeutics that mediate neuronal UBE3A re-expression. The panel strongly recommends that the same or similar test batteries also be employed in the testing of therapeutics directed toward targets downstream of UBE3A, to provide proof-of-concept that the intended biological pathways are being engaged. Going forward, novel behavioral tasks should be developed in AS mice, AS rats, and large animal AS models for the preclinical assessment of therapeutic efficacy in cognitive domains—both to better define critical periods for *UBE3A* re-expression and to more thoroughly vet the safety and brain biodistribution of novel treatments.

### Cognitive functions in AS rodent models

It is of the utmost importance to determine if prolonged *UBE3A* re-expression is required to yield cognitive therapeutic benefits in AS individuals and if these gains can still be achieved if treatment commences during later postnatal life. However, such therapeutic insights into the treatability of AS cognitive deficits could remain elusive without validated tasks to reliably probe cognition in AS animal models. The cognitive deficits thus far described for AS mice have either been variable, thus requiring very large sample sizes to appropriately power analyses, or demand extensive training over many weeks (e.g., operant extinction learning [16]). One solution is to invest in developing high-throughput behavioral tasks for AS mice that reveal robust cognitive phenotypes and are minimally confounded by co-occurring motor deficits. An alternative option is to pursue cognitive testing in the more recently developed AS rat model [17], in which cognitive deficits may be more penetrant and quantifiable. Of course, cognitive tests for AS rodents should be repeatedly validated by multiple independent groups before being relied upon to vet the efficacy of new therapeutics.

### Insights from large animal AS models

Although studies in AS mice strongly suggest that early life treatment will be maximally beneficial to AS individuals [18, 19], much is still unknown regarding critical periods for *UBE3A* re-expression in humans. This knowledge gap persists mainly because postnatal neurodevelopment is very rapid in rodents, with most major milestones completed within 2-3 weeks. Because UBE3A re-expression mediated by either ASOs or gene addition can take several days to reach peak re-expression, it is difficult to precisely evaluate treatment efficacy based on the developmental timing of treatment onset in mice and rats. In contrast, treatment studies utilizing large animal AS models with more protracted postnatal developmental trajectories, including pigs [20] and, perhaps eventually, non-human primates (NHPs), could accommodate more meaningful, albeit still limited [21], extrapolations to human critical periods for *UBE3A* re-expression. Both AS pigs and NHPs could offer additional opportunities to model and overcome challenges inherent to achieving widespread therapeutic delivery and treatment efficacy in a large mammalian brain. Furthermore, NHPs, being uniparous like humans, would provide a more suitable *in utero* system for modeling the safety and efficacy of prenatally administered gene therapies that are irreversible and thus require additional safeguards.

## Advancements for neuronal *UBE3A* re-expression therapeutics

Neuronal *UBE3A* re-expression can be achieved through *UBE3A* gene addition or paternal *UBE3A* unsilencing approaches that leverage small molecules, antisense oligonucleotides (ASOs), CRISPR/Cas systems, or other *UBE3A-ATS*-downregulating technologies [22–32]. Over the past decade, enormous efforts have been made to vet these potential therapeutics using AS mice. Time and again, neuronal *UBE3A* re-expression, regardless of modality, has proven to rescue highly penetrant and AS-relevant phenotypes. So, what stands in the way of translating these preclinical successes in AS mice into a transformative treatment? The convened panel of experts agrees that by closing the following clinical knowledge gaps, we will maximize opportunities to advance therapeutics that mediate neuronal *UBE3A* re-expression in AS individuals.

### Defining genotypic inclusion/exclusion criteria for clinical trials

In addition to other crucial factors including age at trial enrollment and comorbid conditions, genotype must be carefully considered during patient selection for AS clinical trials. Unequivocally, clinical trial candidates should be genetically verified to have AS and a genotype amenable to the therapeutic under study; but for some individuals with *UBE3A* missense mutations [33, 34], this is difficult and time-consuming to confirm. Ideally, a battery of biochemical assays would be developed to identify and exclude *UBE3A* gain-of-function mutations that do not cause AS and potential dominant-negative *UBE3A* variants that are predicted to negate the benefits of paternal *UBE3A* unsilencing. In addition to biochemical assays, rapid and affordable readouts of UBE3A enzymatic dysfunction at the cellular level—e.g., dysregulated target, neurochemical, morphological, or electrophysiological signatures of UBE3A loss of function versus UBE3A gain of function in patient iPSC-derived neurons or other transfected cell lines—could be identified to screen and appropriately categorize *UBE3A* missense variants and, further, serve as preclinical or clinical biomarkers for target engagement.

Individuals with either uniparental disomy (UPD) [1, 35, 36] of the 15q11-q13 region or imprinting center defects (ICD) [1, 37] were initially ineligible to participate in ongoing ASO trials, but more recently have begun to be included in these clinical studies (NCT07157254). The treatment of UPD and ICD individuals with *UBE3A-ATS*-downregulating agents raises concerns about the level of *UBE3A* overexpression that might occur as a consequence of activating two dormant paternal *UBE3A* copies in neurons [38]. Critical insights into the safety and tolerability of *UBE3A* overexpression, as weighed against the potential treatment benefits of neuronal *UBE3A* re-expression for AS individuals, will be garnered from these groups.

### Understanding the impact of other deleted genes in the 15q11-q13 region

The extent to which *UBE3A* re-expression benefits individuals with large 15q11-q13 deletions compared to individuals with deletions or mutations confined to *UBE3A* alone is unclear. Studies of patient-derived neurons and new large-deletion AS mouse models will likely be necessary to elucidate how the haploinsufficiency of other 15q11-q13 genes combines with neuronal *UBE3A* loss of function to impact AS pathogenesis and response to treatment.

### Identifying sensitive and appropriate outcome measures

Sensitive and objectively quantifiable outcome measures would better enable the detection of subtle clinical improvements in AS individuals if they are appropriately matched to a treatment trial with respect to the intervention age, treatment duration, biodistribution of *UBE3A* re-expression, and other treatment variables. For example, no matter how sensitive and quantifiable, cognitive measures would be a poor choice for the primary outcome of a treatment trial in which the biodistribution of *UBE3A* re-expression is biased to the spinal cord. Moreover, given the heterogeneity in clinical presentation among AS individuals across phenotypic domains, baseline metrics must be reliably documented to ensure that phenotypic improvements are detected over the course of treatment. It is of the utmost importance to increase attention and resources for the investigation of AS clinical phenotypes that are widely appreciated to be critical to the quality of life for AS individuals and their families, yet also historically understudied (e.g., communication and sleep), to ensure that they too can be objectively quantified at baseline and in response to treatment. The consensus from the panel of experts was that sensitive measurements relevant to motor, sleep, EEG, and communication phenotypes in AS individuals should ultimately be amenable to objective recording—particularly through the use of wearable devices—in both clinical and in-home settings. Improvements in this area are rapidly emerging and may be critical to demonstrating the therapeutic efficacy of first-generation AS therapeutics in a clinical trial setting. The hope is that optimized next-generation treatments will alleviate AS phenotypes to a much greater extent, thereby obviating the need for exquisitely sensitive outcome measures.

### Establishing measures of UBE3A target engagement

There are no treatment biomarkers to accurately read out UBE3A levels in the brain, only proxies like normalized EEG delta power for which well-calibrated correlations to levels of neuronal *UBE3A* re-expression are lacking [39]. An ideal biomarker of neuronal *UBE3A* re-expression would enable convenient, non-invasive measurements of UBE3A directly in the brain. PET imaging would fit this bill if a suitable UBE3A-sensitive radioligand were to be developed. CSF measures of UBE3A, UBE3A substrates, or other proteins whose levels reliably reflect neuronal UBE3A expression or UBE3A ubiquitin ligase activity may offer other tractable approaches and are justifiable when intra-CSF routes of drug administration are being utilized. Two independent studies have reported the detection of UBE3A in the CSF of AS rats and AS individuals at much lower levels than wild-type rats and neurotypical controls, respectively [40, 41]. Further research is essential to determine if CSF measures of UBE3A can be made repeatedly, reliably, and with sufficient sensitivity to accurately gauge treatment response in AS individuals. Whether this is possible may depend on whether UBE3A in the CSF primarily originates from neurons versus other brain cell types including astrocytes, microglia, or endothelial cells. Non-CSF measures of UBE3A or UBE3A substrates could be considered if (1) the biomarker can be confirmed as brain-derived (e.g. via extracellular vesicles or a brain-specific protein or RNA) or (2) if a relationship between a peripheral biomarker and a brain-specific one can be established in a preclinical model.

### Defining the *UBE3A* re-expression threshold for therapeutic benefit

Correlating biomarker indices with quantifiable phenotypic improvements will help to define *UBE3A* re-expression thresholds for therapeutic benefit, which, in turn, can inform the design and optimal adjustment of dosing regimens for ASOs or other paternal *UBE3A* unsilencers. It should be noted that in contrast to PET biomarkers, most CSF and non-CSF treatment biomarkers would be best suited to reporting mean levels of *UBE3A* re-expression across the brain while offering little insight into the percentage and regional distribution of neurons responding to treatment or the degree of *UBE3A* re-expression per neuron and region. This essential complementary information for CSF and non-CSF biomarkers must be gained from detailed anatomical studies in animal models.

### Determining requirements for sustained UBE3A re-expression

It is unknown if neuronal *UBE3A* re-expression realized during critical periods of early life must be sustained into and throughout adulthood to achieve permanent therapeutic benefits. It is also unclear if certain AS phenotypes are refractory to amelioration unless neuronal *UBE3A* re-expression is established for several months or years. These knowledge gaps are among the most difficult to address through the study of AS animal models and yet have important and obvious implications for dosing strategies for transient treatment modalities (e.g., ASOs, small molecule unsilencers) and the selection of clinical trial endpoints.

## Challenges facing AS gene addition and editing therapies

Phase 3 clinical trials for *UBE3A-ATS*-targeting ASOs are already underway, whereas therapeutics leveraging *UBE3A* gene addition or editing are lagging but beginning to gain ground, with the first phase 1/2 study having commenced at the end of 2025 (NCT07181837).

### The permanence of “one and done” treatments

Whereas ASO and small molecule treatments necessitate daily medication or repeated injections for the lifetime of the patient, one-time delivery of adeno-associated viral vector (AAV) packaged payloads—*UBE3A-ATS*-targeting CRISPR/Cas systems, siRNAs, or functional *UBE3A* transgenes—hold promise for lifelong neuronal *UBE3A* re-expression. However, the perdurance and consequent irreversibility of AAV-based treatments has notable downsides. Dosing cannot be iteratively adjusted for AAV-mediated treatments as it can with ASOs and small molecules and, furthermore, the adaptive humoral immunity evoked by AAVs precludes recipients from receiving a second, potentially more efficacious next-generation AAV therapy. Thus, extra care is needed to vet the safety and efficacy of virally-delivered therapeutics to ensure that a well-tolerated efficacious dose is administered the first and only time.

### The challenge of efficient brain-wide AAV delivery

Another drawback of AAV-based treatments for AS is that, compared to ASOs, capsids based on natural serotypes have limited biodistribution across the central nervous system (CNS) in primates, including humans. On the one hand, limited CNS reach may translate to reduced therapeutic benefit in AS individuals regardless of the route of viral administration. On the other hand, even modest increases in UBE3A activity could significantly mitigate disease phenotypes, as suggested by studies of AS individuals with mosaic ICD [42, 43]. Improved understanding of the requirements for neuronal transduction efficiency within specific brain regions to effect improvement in specific AS phenotypes could enable more reliable estimates of risk versus benefit for AAV-based therapeutics. Moreover, new AAV capsids are emerging that have been selectively engineered to enhance both the spread and efficiency of neuronal transduction throughout the CNS. For example, capsid technologies that exploit transferrin receptor binding to promote AAV transport across the blood-brain barrier (BBB) hold potential for improving the CNS biodistribution of systemically administered AAVs [44]. However, caution is warranted in light of the reported patient death in a clinical trial using a BBB–penetrant engineered AAV capsid from Capsida Biotherapeutics, highlighting a possible discrepancy between safety signals in nonhuman primates and those observed in humans. Further elucidation of the mechanisms underlying the enhanced BBB penetrance of next generation AAV capsids, along with rigorous characterization of their safety profiles in humans, will be essential to advance these emerging technologies.

### Unique challenges for AAV-mediated *UBE3A* gene addition

Paternal *UBE3A* unsilencing is a leading AS therapeutic strategy, in large part because available data indicate that the paternal and maternal *UBE3A* gene copies harbor the same promoter and regulatory elements, thereby obviating concerns for *UBE3A* overexpression or the aberrant expression of UBE3A isoforms in most AS individuals. *UBE3A* gene addition approaches, on the other hand, must employ vector designs that overcome AAV packaging constraints to govern the appropriate proportionality and levels of the three UBE3A isoforms. Recent preclinical efforts have featured dual-isoform vectors that recapitulate expression of the two predominant UBE3A isoforms according to endogenous ratios observed in mouse and human neurons [8]. Additional preclinical research and clinical observations of ASO-treated UPD and ICD individuals (discussed above) will shed light on the impact that overexpression due to *UBE3A* gene addition may (or may not) have on neuronal function. Next-generation vector designs may incorporate feedback mechanisms to safeguard against *UBE3A* overexpression as necessary.

## Alternative and adjunctive therapies

Our panel of experts agreed that *UBE3A* re-expression approaches offer the greatest opportunity for a holistic treatment of AS, but at the same time urged caution not to discount the potential value of alternative treatment strategies, even if they only target a single phenotypic domain. The success of antiepileptic medications in AS illustrates this point: most individuals with AS can achieve acceptable seizure control or seizure freedom with antiseizure medications [45, 46], yet many have treatment-resistant epilepsy that severely compromises their quality of life [46, 47]. Greater insight into epilepsy mechanisms in AS is needed. By cultivating a deep understanding of how UBE3A’s ubiquitination substrates and downstream molecular pathways underpin specific phenotypes, we may open the door to identifying novel, hopefully treatable, alternative AS therapeutic targets [9].

Just like antiepileptic medications, alternative AS therapies can be used adjunctively with *UBE3A* re-expression to mitigate phenotypic effects. This may be an especially effective strategy to deploy in the treatment of older AS individuals when an adjunctive therapy has demonstrated efficacy in mitigating a phenotype beyond the closure of its critical period for *UBE3A* re-expression. Drugs acting as negative or positive effectors of UBE3A enzymatic activity could also be used adjunctively with *UBE3A* re-expression to great effect [48]. For example, suppressors of UBE3A enzymatic activity would be useful for correcting instances of *UBE3A* overdosage in individuals treated with gene addition therapies or in UPD and ICD individuals treated with *UBE3A* unsilencers. Conversely, positive UBE3A effectors could enhance therapeutic benefits when *UBE3A* re-expression occurs at lower than desired levels.

## Concluding remarks

The panel identified many scientific gaps of knowledge that have hindered the development of disease-modifying AS treatments, but also suggested that these gaps are solvable. The importance of gaining deeper understanding of UBE3A function and optimizing therapeutic strategies was emphasized, and specific research priorities were identified to help guide funding agencies and patient-advocacy groups. The ultimate goal for AS, and rare diseases more broadly, is to rapidly, but safely, achieve therapeutic success through synergistic fundamental and clinical research.

## Data Availability

No datasets were generated or analysed during the current study.
